# More precise prediction in Chinese patients with penile squamous cell carcinoma: protein kinase CK2α catalytic subunit (CK2α) as a poor prognosticator

**DOI:** 10.18632/oncotarget.17935

**Published:** 2017-05-16

**Authors:** Zai-Shang Li, Chuang-Zhong Deng, Yun-Lin Ye, Kai Yao, Sheng-Jie Guo, Jie-Ping Chen, Yong-Hong Li, Zi-Ke Qin, Zhuo-Wei Liu, Bin Wang, Qi Zhao, Peng Chen, Qi-Wu Mi, Xiao-Feng Chen, Hui Han, Fang-Jian Zhou

**Affiliations:** ^1^ Department of Urology, Sun Yat-sen University Cancer Center, Guangzhou, P. R. China; ^2^ State Key Laboratory of Oncology in Southern China, Guangzhou, P. R. China; ^3^ Collaborative Innovation Center of Cancer Medicine, Guangzhou, P. R. China; ^4^ Department of Urology, Cancer Center of Guangzhou Medical University, Guangzhou, P. R. China; ^5^ School of Life Science, Sun Yat-sen University, Guangzhou, P. R. China; ^6^ Department of Urology, Affiliated Tumor Hospital of Xinjiang Medical University, Urumchi, P. R. China; ^7^ Department of Urology, Dong Guan People's Hospital, Guang Dong, P. R. China; ^8^ Department of Urology, The First People's Hospital of Chenzhou, Chenzhou, P. R. China

**Keywords:** casein kinase 2α, penile neoplasm, squamous cell carcinoma

## Abstract

**Purpose:**

In this study, we assess the CK2α expression in human penile squamous cell carcinoma (SCC) and its clinical significance.

**Methods:**

A total of 157 human penile SCC tissue samples were immunohistochemically analyzed. In addition, 12 human penile SCC and adjacent normal tissues were examined for CK2α protein and mRNA expression by Western blotting and real-time quantitative PCR, respectively. Survival was analyzed using the Kaplan-Meier test and the log-rank test. Multivariate Cox proportional hazard regression analysis was performed to determine the impacts of CK2α expression and the clinicopathological features on patient disease-specific survival (DSS). Likelihood ratios (LRs), Akaike information criterion (AIC) values, and concordance indexes (C-indexes) were investigated to evaluate the accuracies of the factors. Bootstrap-corrected C-indexes were used for internal validation (with sampling 1000 times).

**Results:**

A significant difference in the distribution of CK2α was observed between the normal and penile carcinoma tissues (*P*<0.001). CK2α expression was associated with the pathological T and N stages in the penile cancer tissues (*P*<0.001). High CK2α expression was with significantly poorer DSS compared with low expression one (*P*<0.001). Western blotting and real-time quantitative PCR also confirmed that CK2α expression was increased in the penile cancer tissues. In multivariate Cox regression analysis, CK2α overexpression still was one of independent prognostic factors for penile SCC (*P*=0.005). The predictive accuracy of CK2α was verified by analysis of the C-indexes.

**Conclusion:**

High protein kinase CK2α expression is associated with several prognostic factors and is thus a significant indicator of poor prognosis for penile cancer.

## INTRODUCTION

Penile cancer is a rare tumors in the Europe and United States, with an incidence of less than 1.00 per 100,000 males [1]. However, it is still a major public health problem in non-Western countries, such as Uganda, Brazil, and India [2, 3]. The main pathology type( ≥95%) of penile cancer are squamous cell carcinomas (SCCs), and the lymph node metastasis (LNM) are considered as the most important predictors of survival [2]. In contrast with other genitourinary cancers, penileSCC is typically characterized by stepwise regional lymph node (LN) spread, which is clearly related to survival [1, 2, 4, 5]. Nevertheless, the outcomes vary among patients with pathologically proven nodal disease. In addition, the survival rates of these patients exhibit large variations according to the extent of nodal involvement [2, 4, 6]. Thus, the detection and treatment of these metastases as soon as possible and improved prediction of survival are important for clinicians managing these patients.

The stage, grade of differentiation and clinicopathological factors of a primary tumor are well known to be predictive factors for LNM and survival [2, 6, 7]. However, deciding to perform regional node dissection based solely on these characteristics leads to unacceptable false-negative and false-positive rates. Inguinal lymph node dissection (ILAD) alone is curative in many cases with minimal metastases [2–4, 6, 7]. Nevertheless, a considerable number of men with palpable nodes do not experience metastatic spread, and consequently, up to 80% may be overtreated [8, 9], with resulting high morbidity [10, 11]. However, following a wait-and-see policy in treatment of these patients poses the risk of metastasis development to a stage at which a cure is no longer possible [12]. Thus, increased understanding of the molecular factors and biological features is of paramount importance in penile SCC [13, 14].

Protein kinase CK2 is a highly conserved and ubiquitous serine/threonine kinase composing with two catalytic subunits and two regulatory subunits [15]. CK2 is as a multifunctional protein kinase that is widely distributed in substrates [16, 17]. Disruption of *Saccharomyces cerevisiae* genes encoding CK2 catalytic subunits is a lethal event, and it plays major roles in cell cycle progression, proliferation, and differentiation processes [18, 19]. In addition, the clinical significance of CK2α subunits have been assessed in human cancers [20–24]. Thus, CK2α is now considered a potential target in the treatment of human malignancy [25]. As we’ve seen, the prognostic significance of nuclear CK2α in penile SCC is still unknown.

In this pilot study, we evaluated CK2α expression in primary penile SCC. In addition, the relationships between CK2α expression and clinicopathologic features were examined, as well as the clinical prognostic value of CK2α in penile SCC.

## RESULTS

After a median time of 25 mo (range 1–133 mo), 43 patients died of penile cancer. The patients’ clinical characteristics are shown in Table 1.

**Table 1:** CK2α expression in penile SCC and its correlation with the clinicopathologic parameters

Variable	No. (%) or variable unit	CK2α expression		*P*	DSS		*P*
		Negative	Positive		3 years	5 years	
Age (Year)	52(24-82)			0.160			0.403
<52	78(49.7)	32(40.0)	48(60.0)		74.5(64.1-84.9)	71.6(60.0-83.2)	
≥52	79(50.3)	22(28.6)	55(71.4)		67.9(56.3-79.5)	58.5(43.2-73.8)	
BMI (kg/m^2^)	22.6(14.2-36.7)			0.911			0.686
<22.6	78(49.7)	26(33.3)	52(66.7)		70.2(59.2-81.2)	63.2(49.7-76.7)	
≥22.6	79(50.3)	27(34.2)	52(65.8)		73.9(62.7-85.1)	67.7(54.6-80.8)	
T No. (%)				0.001			<0.001
≤pT1	48(30.6)	26(54.2)	22(45.8)		89.8(78.6-100.0)	89.8(78.6-100.0)	
≥pT2	109(69.4)	27(24.8)	82(75.2)		64.4(54.8-74.0)	54.1(41.6-66.6)	
N No. (%)				<0.001			<0.001
N0	74(47.1)	40(54.1)	34(45.9)		90.3(82.7-97.9)	82.7(70.2-95.2)	
N+	83(52.9)	13(15.7)	70(84.3)		55.7(43.7-67.7)	49.5(36.8-62.2)	
G No. (%)				0.177			0.022
G1	92(58.6)	35(38.0)	57(62.0)		75.7(65.9-85.5)	75.7(65.9-85.5)	
≥G2	65(41.4)	18(21.5)	47(78.5)		67.4(55.1-79.7)	51.1(34.0-68.2)	

**Figure 1:**
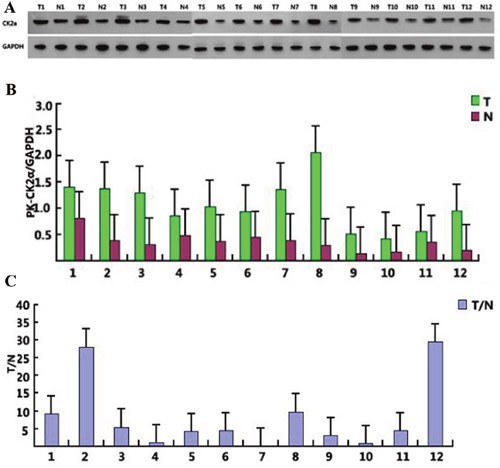
CK2α expression in 12 selected tumor samples CK2α levels were measured by Western blotting and qPCR **(A** and **B)** and the mRNA levels were then compared between normal and cancer penile tissues **(C)**.

**Figure 2:**
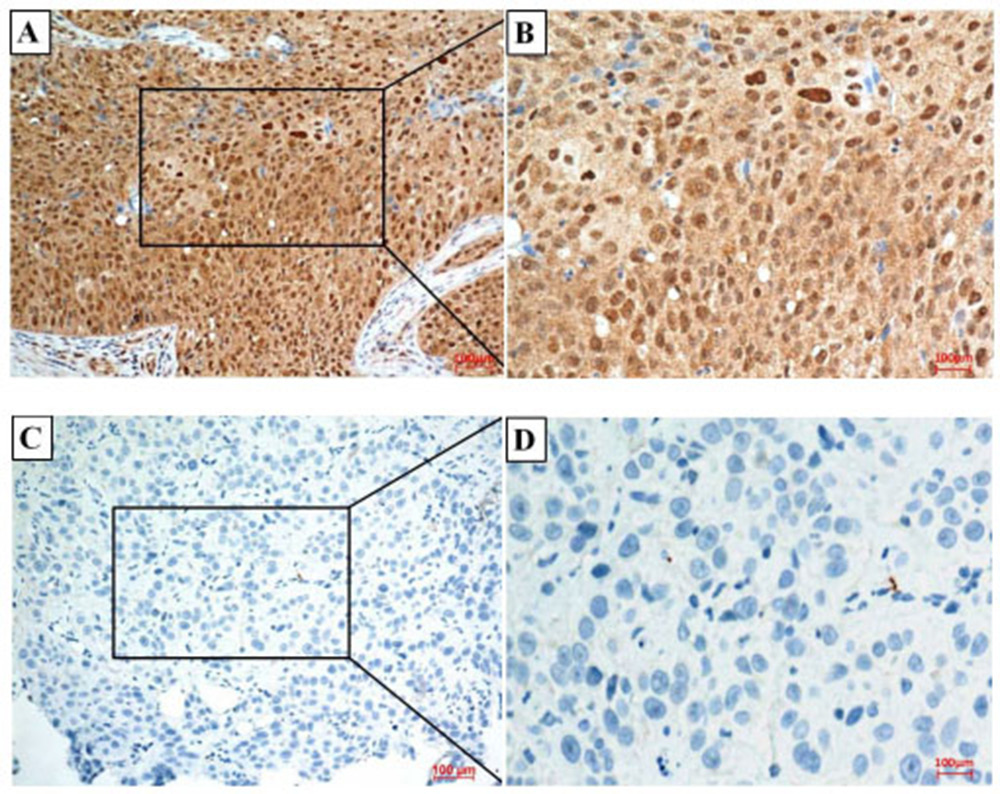
CK2α expression in penile cancer tissues Negative and positive results are shown in Panels **(A**-**D)**, respectively. **(A)** A positive of CK2α expression in penile cancer tissues (200×); **(B)** 400× magnification of the boxed area shown in **(A)**. **(C)** A negative of CK2α expression in penile cancer tissues (200×); **(D)** 400× magnification of the boxed area shown in **(C)**.

Immunohistochemical analysis revealed that CK2α was expressed in both the cell nucleus and cytoplasm (Figure 1A, 1B), and cancer tissues were with high CK2α expression. Similar to the immunohistochemistry results, CK2α expression was significantly higher in the penile cancer tissues in 12 normal-tumor tissue pairs by Western blotting (Figure 2A, 2B) (*P*<0.001). Additionally, quantitative real-time PCR results also proved that CK2α expression was significantly increased in the tumor tissues over the non-tumor ones (Figure 2C).

These results indicated that CK2α expression was significantly correlated with the T stage (*P*=0.001) and N status (*P* < 0.001). However, there was no significant association between nuclear CK2α expression and age, body mass index (BMI), or tumor grade. The associations between CK2α immunoreactivity and the clinicopathologic features are shown in Table 1.

**Figure 3:**
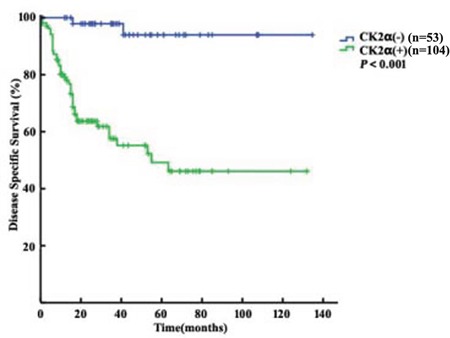
Disease Specific Survival analysis of 157 penile cancer patients stratified by CK2a immunoreactivity (positiveCK2α: final staining score of ≥6 ;negative CK2α: final staining score of <6) All statistical tests were two-sided. Significance level: P<0.001.

Table 1 showed that DSS was significantly associated with CK2α overexpression (*P* < 0.001). The patients with higher CK2α expression had 3-year and 5-year DSS rates of 57.6% (46.4%-68.8%) and 49.2% (36.3%-62.1%), compared with rates of 97.9% (93.8%-100.0%) and 94.0% (85.6%-100.0%) in the patients with low CK2α expression (*P* < 0.001, Figure 3).

The results of univariate analyses related to DSS are shown in Table 1. They indicated that the T stage and N status (all *P* < 0.001), tumor grade (*P*=0.022) and CK2α overexpression (*P*<0.001) were significantly correlated with DSS. Further, Cox multivariate analysis revealed that CK2α overexpression was an independent prognostic predictor (Table 2, *HR*=8.234, *95% CI*=1.912-35.460*, P*=0.005). CK2α overexpression showed the best predictive accuracy for the LR, C-index and AIC among the basic factors. Accounting for CK2α expression, the predictive accuracy of the basic factors were with significantly increased (Table 3).

**Table 2:** Multivariate Cox regression analyses for DSS

Variable	Multivariate analysis model		
	HR	95% CI	*P*
Age (age)			
<52 vs ≥52	1.522	0.818-2.833	0.185
BMI (kg/m^2^)			
<22.6 vs ≥22.6	0.693	0.376-1.275	0.238
T			
≤T1 vs ≥T2	5.335	1.615-17.625	0.006
N			
N0 vs N+	2.534	1.127-5.699	0.025
G			
G1 vs ≥G2	1.319	0.710-2.450	0.369
CK2α expression			
Negative vs Positive	8.234	1.912-35.460	0.005

**Table 3:** Predictive accuracy of factors

Variable	LR*	AIC	C-index	Bootstrap C-index
T (≤T1 vs ≥T2)	18.604	169.740	0.662	0.667
N (N0 vs N+)	20.631	167.710	0.696	0.702
G (G1 vs ≥G2)	5.021	183.320	0.599	0.597
CK2α expression(negative vs positive)	27.827	160.520	0.700	0.699
T+CK2α expression	38.675	151.670	0.776	0.777
N +CK2α expression	36.378	153.970	0.766	0.765
G+CK2α expression	31.307	158.870	0.750	0.750

*Logistic, AIC=Akaike information criterion, LR=likelihood ratio, C-index=concordance index.

## DISCUSSION

CK2α expression has been assessed in a part of turmors, including prostate, colorectal and lung cancers [20–24]. However, with limited clinical data, the clinical significance of CK2α hasn't been studied. In the pilot study, we investigated the expression of CK2α in penile tissues from patients with penile cancer.

To our knowledge, CK2α expression in human penile tissues is unknown, and it has not yet been measured in human penile cancer. In the present study, significant differences in CK2α expression were detected between penile cancer tissues and non-tumor tissues (Figure 2). Several reports have consistently demonstrated that the CK2 level are increased in a variety of cancers [22, 23]. A recent study has shown that nuclear CK2α expression in tumor colorectal tissues was significantly increased compared with that in non-tumor colorectal ones [22]. Another previous study involving cancer has demonstrated that CK2α accumulates in cancer cells and plays a key role in promoting cell survival [20]. Some studies considered that increasing CK2α expression might be related to the increased proliferative capacity of tumor cells [25–28].

For the first time, the results of our study showed that CK2α overexpression was closely correlated with the pathologic tumor status (*P*=0.001) and pathologic nodal status (*P*<0.001) (Table 1). Although the mechanisms underlying these associations of CK2α are still unclear, these correlations are supported by several current lines of evidence. Mottet et al. have shown that blocking of the activation of hypoxia-inducible factor-1 (HIF-1) leads to CK2 overexpression and subsequent VEGF-C inhibition [29]. In addition, HIF-1 has been shown to stimulate the expression of VEGF-C, which is associated with the development of LNM [30]. Timofeeva et al. have reported that signal transducers and activators of transcription (STATs) which plays a critical role in Wilms’ tumor are downstream targets of CK2 [31]. Constitutively, activation of STATs, which play the key role in the growth and differentiation, facilitate neoplastic behaviors of a number of cancers [32, 33]. Yao et al. have showed that CK2 activity suppresses cell proliferation and even induces cell death in both LNCaP and PC-3/AR human prostate cancer cells compared with mock-transfected control cells [21]. As a CK2 catalytic subunit, CK2α may play an important role in the marked resistance of cancer cells to apoptotic signals. This array of cellular responses is broadly orchestrated by CK2-dependent regulation of key signaling molecules in the Hedgehog/Gli, NF_K_B, Wnt, PI3K/AKT/mTOR and hypoxia pathways that have been multifunction [34–37].

Our study also demonstrated that CK2α expression can function as an independent of prognostic markers for penile cancer (Tables 2 and 3). However, clinical data pertaining to the clinic value of CK2α are limited. Laramas et al. had shown that CK2α protein expression is elevated in prostate cancer by immunohistochemical analysis only [38]. In 2011, Lin et al. demonstrated that overexpression of nuclear CK2α in colorectal cancer tissues was correlated with several clinicopathologic factors and that it could be used as an independent prognostic marker for colorectal cancer [22]. Further, a study in which global gene expression profiling was performed on 190 human tumors of 14 cancer types resulted in the identification of 11 genes, including CK2, and the results suggested that the expression of CK2 was strongly correlated with that of MT1-MMP, in direct association with tumor prognosis [39]. However, no report of the prognostic significance of CK2α in penile SCC has done. Importantly, our results showed that CK2α overexpression could be used as an independent prognostic marker for penile cancer. In addition, accounting for CK2α expression in multivariate analysis of the basic factors had a positive impact on predictive accuracy (Table 3).

In summary, this pilot study revealed that high CK2α expression was significantly associated with prognosis in penile cancer treated with surgery. Thus, patients with penile cancer exhibiting CK2α overexpression should be carefully monitored.

## PATIENTS AND METHODS

### Patients

After being approved by the institutional review board, patients with penile carcinoma treated at Sun Yat-sen University Cancer Center from March 2002 to March 2013 were retrospectively analyzed. A total of 43 patients presented with neoadjuvant chemotherapy and/or radiotherapy, and 87 presented with metastatic disease. Patients were excluded if they had received palliative therapy or if their pathological data were incomplete. Ultimately, 157 patients with bilateral ILND were included in the study population.

The non-tumor tissues were obtained by separation of the grossly normal tissues from the tumor tissues in the resected specimens. After uropathological review, the pathologic features were determined anew according to the AJCC 7th TNM staging system. The histological grade was classified according to the Broder system. Long term follow - up: every 3 month for the first 2 yr after surgery, every 6 mo during the 3rd -4th years, and then once yearly thereafter. The follow-up deadline was December 2015.

### Immunohistochemistry and interpretation

Mastadenoma and omission of the primary antibody were used as positive and negative control. 3% H_2_O_2_ was using for blocking endogenous peroxidase for 10 min, and 10% normal goat serum was using for blocking nonspecific binding of immunoglobulins for 10 min. With appropriately diluted primary antibodies, sections were placed at 4°C for one night. The slides were subsequently incubated with a primary antibody, clonal anti-CK2α (Millipore, Massachusetts, USA 1:50 dilution) at 25°C for 30 minutes. After 5-8 minutes incubation with 3,3′-diaminobenzidine, the sections were counterstained with hematoxylin and microscopic analysis.

Two pathologists was independently scored immunoreactivity. Nuclear and cytoplasmic staining were both scored for the sake of the ubiquitous and nucleo-cytoplasmic distribution of CK2α. According to the percentage of positive staining area, the score were 0 (0%), 1 (1-20%), 2 (21-50%), 3 (51-75%), and 4 (76-100%). And the following scores were used: 0 = no staining; 1+ = weak staining; 2+ = moderate staining; and 3+ = strong staining. The sum of the intensity and extent scores range 0 to 7. For the final statistics, tumors with score of ≥6 were considered positive (+) for CK2α. All other tumors were considered negative (-).

### Quantitative real-time PCR

12 tumor and non-tumor pairs of colorectal tissues using for RNA with an RNA extraction kit (TAKARA, Dalian, China). RNA quality was analyzed using an Agilent Stratagene Bioanalyzer (Palo Alto, California, USA). The RNA integrity numbers (RINs) of all 24 samples indicated that the RNA quality was good, and the samples were then used for cDNA synthesis, after which they were stored at -20°C until further use. The amplification mixture (25 μl) contained 0.030 ng cDNA (3 μl), 1× TaqMan Master Mix, and 1× TaqMan Gene Expression Assay Primer/Probe Mix (TAKARA, Dalian, China). The thermal cycling parameters were as follows: 40 cycles at 95°C for 2 min, 95°C for 30 sec, 60°C for 30 sec, and 72°C for 30 s for pre-denaturation, denaturation, annealing and extension, respectively. The sequences of the primers used for PCR amplification designed according to GenBank mRNA sequences were TGTTCGTCATGGGTGTGAAC (forward) and ATGGCATGGACTGTGGTCAT (reverse).

### Western blot

The mixture of protease and phosphatase inhibitors (50 mM Tris-HCl, pH 7.5, 1% Triton X-100, 150 mM NaCl, 1 mM NaF, 1 mM Na_3_VO_4_, 10 mM sodium pyrophosphate, 1 mM phenylmethylsulfonyl fluoride, 1 lg/ml aprotinin and 1 lg/ml leupeptin) were useing for protein extracts. Extracted protein should be centrifugated at 14,000 g to remove debris. Denatured proteins in the samples were subjected to 10% SDS-PAGE. Anti-CK2α polyclonal antibody (1:1500 dilution) and phosphate-buffered serum (PBS:5% milk +0.05% Tween-20) was using for blocking at 25°C room temperature overnight. GAPDH (Sigma, 1:1500 dilution) was used as an internal control. Subsequently, goat anti-rabbit antibody (1:5000 dilution) was using for incubation for 1 hour at 25°C room temperature. Targeted proteins was enhanced with chemiluminescence reagents (Millipore). All experiments were conducted three times independently.

### Statistical analysis

Receiver operating characteristic (ROC) curves were generated to identify the best point for prediction of patient prognosis. The correlations between CK2α expression and the clinicopathologic parameters were examined using Pearson's correlation analysis. DSS was calculated using the Kaplan-Meier method, and assessed by log-rank tests. Univariate and multivariate Cox proportional hazard regression analyses were performed to determine the impacts of the clinical and pathological factors on patient DSS with removal of nonsignificant factors in a stepwise backward fashion. Likelihood ratios (LRs), Akaike information criterion (AIC) values, and concordance indexes (C-indexes) were investigated to evaluate the accuracies of the factors. Bootstrap-corrected c-indexes were used for internal validation to better gauge expected future predictive accuracy (with sampling 1000 times). All data were analyzed using R2.11.1 (http://www.r-project.org), with statistical significance set at P<0.05.

## References

[1] Barnholtz-Sloan JS, Maldonado JL, Pow-sang J, Giuliano AR (2007). Incidence trends in primary malignant penile cancer. Urol Oncol.

[2] Hakenberg OW, Comperat EM, Minhas S, Necchi A, Protzel C, Watkin N (2015). EAU guidelines on penile cancer: 2014 update. Eur Urol.

[3] Misra S, Chaturvedi A, Misra NC (2004). Penile carcinoma: a challenge for the developing world. Lancet Oncol.

[4] Brady KL, Mercurio MG, Brown MD (2013). Malignant tumors of the penis. Dermatol Surg.

[5] Leijte JA, Valdes OR, Nieweg OE, Horenblas S (2008). Anatomical mapping of lymphatic drainage in penile carcinoma with SPECT-CT: implications for the extent of inguinal lymph node dissection. Eur Urol.

[6] Edge SB, Compton CC (2010). The American Joint Committee on Cancer: the 7th edition of the AJCC cancer staging manual and the future of TNM. Ann Surg Oncol.

[7] Solsona E, Iborra I, Rubio J, Casanova JL, Ricos JV, Calabuig C (2001). Prospective validation of the association of local tumor stage and grade as a predictive factor for occult lymph node micrometastasis in patients with penile carcinoma and clinically negative inguinal lymph nodes. J Urol.

[8] Hegarty PK, Kayes O, Freeman A, Christopher N, Ralph DJ, Minhas S (2006). A prospective study of 100 cases of penile cancer managed according to European Association of Urology guidelines. Bju Int.

[9] Kroon BK, Horenblas S, Lont AP, Tanis PJ, Gallee MP, Nieweg OE (2005). Patients with penile carcinoma benefit from immediate resection of clinically occult lymph node metastases. J Urol.

[10] Spiess PE, Hernandez MS, Pettaway CA (2009). Contemporary inguinal lymph node dissection: minimizing complications. World J Urol.

[11] Yao K, Tu H, Li YH, Qin ZK, Liu ZW, Zhou FJ, Han H (2010). Modified technique of radical inguinal lymphadenectomy for penile carcinoma: morbidity and outcome. J Urol.

[12] Kroon BK, Horenblas S, Estourgie SH, Lont AP, Valdes OR, Nieweg OE (2004). How to avoid false-negative dynamic sentinel node procedures in penile carcinoma. J Urol.

[13] Muneer A, Kayes O, Ahmed HU, Arya M, Minhas S (2009). Molecular prognostic factors in penile cancer. World J Urol.

[14] Zhu Y, Zhou XY, Yao XD, Zhang SL, Dai B, Zhang HL, Shen YJ, Ye DW (2013). Prognostic value of carbonic anhydrase IX expression in penile squamous cell carcinoma: a pilot study. Urol Oncol.

[15] Filhol O, Cochet C (2009). Protein kinase CK2 in health and disease: Cellular functions of protein kinase CK2: a dynamic affair. Cell Mol Life Sci.

[16] Litchfield DW (2003). Protein kinase CK2: structure, regulation and role in cellular decisions of life and death. Biochem J.

[17] St-Denis NA, Litchfield DW (2009). Protein kinase CK2 in health and disease: From birth to death: the role of protein kinase CK2 in the regulation of cell proliferation and survival. Cell Mol Life Sci.

[18] Buchou T, Vernet M, Blond O, Jensen HH, Pointu H, Olsen BB, Cochet C, Issinger OG, Boldyreff B (2003). Disruption of the regulatory beta subunit of protein kinase CK2 in mice leads to a cell-autonomous defect and early embryonic lethality. Mol Cell Biol.

[19] Padmanabha R, Chen-Wu JL, Hanna DE, Glover CV (1990). Isolation, sequencing, and disruption of the yeast CKA2 gene: casein kinase II is essential for viability in Saccharomyces cerevisiae. Mol Cell Biol.

[20] Yao K, Youn H, Gao X, Huang B, Zhou F, Li B, Han H (2012). Casein kinase 2 inhibition attenuates androgen receptor function and cell proliferation in prostate cancer cells. Prostate.

[21] Zhang S, Wang Y, Mao JH, Hsieh D, Kim IJ, Hu LM, Xu Z, Long H, Jablons DM, You L (2012). Inhibition of CK2alpha down-regulates Hedgehog/Gli signaling leading to a reduction of a stem-like side population in human lung cancer cells. PLoS One.

[22] Lin KY, Tai C, Hsu JC, Li CF, Fang CL, Lai HC, Hseu YC, Lin YF, Uen YH (2011). Overexpression of nuclear protein kinase CK2 alpha catalytic subunit (CK2alpha) as a poor prognosticator in human colorectal cancer. PLoS One.

[23] Shimada K, Anai S, Marco DA, Fujimoto K, Konishi N (2011). Cyclooxygenase 2-dependent and independent activation of Akt through casein kinase 2alpha contributes to human bladder cancer cell survival. BMC Urol.

[24] Giusiano S, Cochet C, Filhol O, Duchemin-Pelletier E, Secq V, Bonnier P, Carcopino X, Boubli L, Birnbaum D, Garcia S, Iovanna J, Charpin C (2011). Protein kinase CK2alpha subunit over-expression correlates with metastatic risk in breast carcinomas: quantitative immunohistochemistry in tissue microarrays. Eur J Cancer.

[25] Zhu D, Hensel J, Hilgraf R, Abbasian M, Pornillos O, Deyanat-Yazdi G, Hua XH, Cox S (2010). Inhibition of protein kinase CK2 expression and activity blocks tumor cell growth. Mol Cell Biochem.

[26] Hekmat O, Munk S, Fogh L, Yadav R, Francavilla C, Horn H, Wurtz SO, Schrohl AS, Damsgaard B, Romer MU, Belling KC, Jensen NF, Gromova I (2013). TIMP-1 increases expression and phosphorylation of proteins associated with drug resistance in breast cancer cells. J Proteome Res.

[27] Ruzzene M, Pinna LA (2010). Addiction to protein kinase CK2: a common denominator of diverse cancer cells?. Biochim Biophys Acta.

[28] Kanki T, Kurihara Y, Jin X, Goda T, Ono Y, Aihara M, Hirota Y, Saigusa T, Aoki Y, Uchiumi T, Kang D (2013). Casein kinase 2 is essential for mitophagy. Embo Rep.

[29] Mottet D, Ruys SP, Demazy C, Raes M, Michiels C (2005). Role for casein kinase 2 in the regulation of HIF-1 activity. Int J Cancer.

[30] Semenza GL (1999). Regulation of mammalian O2 homeostasis by hypoxia-inducible factor 1. Annu Rev Cell Dev Biol.

[31] Timofeeva OA, Plisov S, Evseev AA, Peng S, Jose-Kampfner M, Lovvorn HN, Dome JS, Perantoni AO (2006). Serine-phosphorylated STAT1 is a prosurvival factor in Wilms' tumor pathogenesis. Oncogene.

[32] Huang S, Bucana CD, Van Arsdall M, Fidler IJ (2002). Stat1 negatively regulates angiogenesis, tumorigenicity and metastasis of tumor cells. Oncogene.

[33] Hudelist G, Czerwenka K, Singer C, Pischinger K, Kubista E, Manavi M (2005). cDNA array analysis of cytobrush-collected normal and malignant cervical epithelial cells: a feasibility study. Cancer Genet Cytogenet.

[34] Landesman-Bollag E, Song DH, Romieu-Mourez R, Sussman DJ, Cardiff RD, Sonenshein GE, Seldin DC (2001). Protein kinase CK2: signaling and tumorigenesis in the mammary gland. Mol Cell Biochem.

[35] Ji H, Wang J, Nika H, Hawke D, Keezer S, Ge Q, Fang B, Fang X, Fang D, Litchfield DW, Aldape K, Lu Z (2009). EGF-induced ERK activation promotes CK2-mediated disassociation of alpha-Catenin from beta-Catenin and transactivation of beta-Catenin. Mol Cell.

[36] Guerra B (2006). Protein kinase CK2 subunits are positive regulators of AKT kinase. Int J Oncol.

[37] Kallergi G, Markomanolaki H, Giannoukaraki V, Papadaki MA, Strati A, Lianidou ES, Georgoulias V, Mavroudis D, Agelaki S (2009). Hypoxia-inducible factor-1alpha and vascular endothelial growth factor expression in circulating tumor cells of breast cancer patients. Breast Cancer Res.

[38] Laramas M, Pasquier D, Filhol O, Ringeisen F, Descotes JL, Cochet C (2007). Nuclear localization of protein kinase CK2 catalytic subunit (CK2alpha) is associated with poor prognostic factors in human prostate cancer. Eur J Cancer.

[39] Rozanov DV, Savinov AY, Williams R, Liu K, Golubkov VS, Krajewski S, Strongin AY (2008). Molecular signature of MT1-MMP: transactivation of the downstream universal gene network in cancer. Cancer Res.

